# Materials Acceleration Platform for Electrochemistry: a Platform for Autonomous Electrochemistry

**DOI:** 10.1038/s41529-026-00822-8

**Published:** 2026-06-04

**Authors:** Daniel Persaud, Mike Werezak, Mark Xu, Melyne Zhou, Frank Benkel, Xin Pang, Vahid Attari, Brian DeCost, Ashley S. Dale, Nicholas Senior, Gabriel Birsan, Jason Hattrick-Simpers

**Affiliations:** 1 CanmetMATERIALS, Natural Resources Canada, Hamilton, ON Canada; 2 Department of Materials Science and Engineering, University of Toronto, Toronto, ON Canada; 3 Material Measurement Laboratory, National Institute of Standards and Technology, 100 Bureau Dr, Gaithersburg, MD USA; 4 Acceleration Consortium, University of Toronto, Toronto, ON Canada; 5 Vector Institute for Artificial Intelligence, Toronto, ON Canada; 6 Schwartz Reisman Institute for Technology and Society, Toronto, ON Canada

**Keywords:** Chemistry, Engineering, Materials science

## Abstract

Corrosion testing is slow, labor-intensive, and sensitive to operator technique, limiting the generation of large, high-quality datasets for data-driven materials discovery. The Materials Acceleration Platform for Electrochemistry (MAP-E) is an autonomous, high-throughput system, capable of performing parallel electrochemical experiments. It integrates robotic liquid handling and sample transfer with a multi-channel potentiostatic control to extract corrosion metrics without human intervention. Validation against an ASTM G61-analog benchmark demonstrates good reproducibility, with a standard deviation of 75 mV in pitting potential across 32 automated measurements. The platform was then employed to autonomously construct pH-chloride stability diagrams for 304 stainless steel using an uncertainty-driven sampling strategy on a Gaussian process surrogate model. This approach reduces operator involvement and accelerates the exploration of environmental spaces. The MAP-E establishes a framework for autonomous electrochemical experimentation, enabling generation of corrosion datasets that inform materials discovery, alloy design, and durability assessment in service environments.

## Introduction

The corrosion of metals and alloys remains a major challenge in ensuring the safety and reliability of infrastructure, transportation, and energy systems^1–3^. During service, materials are exposed to a diverse range of complex environments that can accelerate degradation, leading to significant safety concerns and as a results, substantial economic cost^2–8^. Predicting how materials will perform under different service conditions, or designing new materials with improved corrosion resistance for specific environments, requires an understanding of the complex interplay between alloy chemistry, microstructure, surface condition, and environmental factors^9–11^. This multifaceted nature makes the systematic exploration of corrosion phenomena experimentally demanding, especially because traditional electrochemical experiments are labor-intensive and sensitive to procedural details, requiring considerable experience to achieve reproducible results^3,12^. As a result, generating large, high-quality datasets that capture corrosion behavior across broad parameter spaces is challenging, limiting the ability to establish predictive relationships between material properties, environment, and degradation mechanisms.

Autonomous electrochemical platforms have the potential to address these challenges by integrating networked instrumentation, modular fluid handling, and parallelized electrochemical cells under unified control software^8,13,14^. When coupled with machine learning (ML), these systems can be used to generate informative datasets through efficient exploration of complex experimental spaces to better understand underlying patterns. The datasets produced can be shared with the broader research community, enabling others to develop predictive models, identify promising research directions, and collaborate to accelerate innovation.

Recent years have seen substantial progress in high-throughput (HT) electrochemical platforms, including systems that integrate hardware and software to varying degrees of autonomous operation. Early HT approaches emphasized parallel screening to maximize throughput, often relying on shared electrolytes, reduced solution volumes, simplified electrode configurations, or measurements limited to post-exposure or optical characterization^15–19^. More recent efforts have shifted toward increased autonomy through integrated hardware-software platforms, commonly based on scanning droplet cells or gantry-based systems that sequentially position a shared reference and counter electrode into electrolyte-filled wells to characterize the sample below^20–29^. Despite these advances, most platforms face trade-offs: systems closer to autonomy often perform experiments serially, limiting speed, whereas parallelized systems frequently compromise on experimental fidelity, restricting their relevance for corrosion science. These accommodations are typically driven by constraints in hardware, cost, or the complexity associated with linking multiple pieces of equipment^30,31^. Collectively, these limitations define a critical gap for corrosion research: the absence of a platform that can perform parallel electrochemical measurements, using traditional cell geometries, electrolyte volumes, and standard electrode configurations, features necessary for high-quality data-driven exploration across diverse material-environment combinations.

To address this gap, we have developed the Materials Acceleration Platform for Electrochemistry (MAP-E), a flexible, HT platform designed for autonomous electrochemical experiments. It integrates eight individually addressable electrochemical flat-cells, each capable of performing independent measurements in parallel. The platform features modular fluid handling to explore a wide range of aqueous environments. Robust component control and scheduling software manages complex experimental campaigns, enabling automated operation. With these capabilities, the MAP-E can generate the precise and reliable electrochemical data required for applying ML algorithms to autonomously explore experimental spaces.

In this work, we first validate the MAP-E’s ability to perform reproducible electrochemical measurements using a modified protocol derived from an established corrosion standard. We then demonstrate its ability to autonomously construct data-driven stability diagrams within a single closed-loop campaign, demonstrating a more efficient use of operator time compared to traditional sequential approaches. Once the stock solutions and samples are prepared, the system loads samples and mixes solution to explore the defined experimental space without further human intervention. By running eight cells in parallel, the MAP-E achieves an immediate eightfold increase in throughput. An uncertainty-driven acquisition strategy is used to adaptively select subsequent experimental conditions based on the model’s posterior predictive uncertainty. This focuses sampling on regions where the model is least certain, allowing informative areas of the design space to be explored efficiently under a constrained experimental budget. By producing standard-aligned, reproducible data and demonstrating the autonomous exploration of experimental spaces, the MAP-E can support collaborative, data-driven materials discovery through the generation and public sharing of high-fidelity experimental datasets.

## Results and Discussion

### Corrosion Validation

As discussed above, the design and configuration of electrochemical platforms vary widely, making comparison across laboratories difficult^14^, and with eight independent cells, it is also necessary to verify intra-platform reproducibility^32^. Accordingly, we aimed to validate the electrochemical performance of the MAP-E using experiments aligned with standards commonly employed in corrosion testing to assess reproducibility. These experiments were performed to demonstrate that the MAP-E can generate reliable electrochemical data suitable for subsequent adaptive experimentation.

To validate the electrochemical performance of the MAP-E, we performed potentiodynamic polarization (PP) experiments using an analog to ASTM G61^33^, a standard for evaluating localized corrosion susceptibility of alloys in aqueous chloride (Cl^−^) environments. Following as closely as possible, the procedure allows us to quantitatively benchmark the MAP-E’s performance against established metrics for pitting potential (*E*_pit_). ASTM G61 defines expected inter-laboratory reproducibility and bias in *E*_pit_ during PP measurements. Based on results from five independent laboratories, the standard reports an inter-laboratory 2*σ* of ≈ 600 mV in *E*_pit_. However, it does not define intra-laboratory repeatability, leaving the expected variability when multiple identical tests are performed within a single laboratory, or in the case of the MAP-E, a single cell, unspecified.

Several adaptations to ASTM G61 were made to accommodate the MAP-E’s capabilities. The platform operates under ambient (aerated) conditions rather than the nitrogen-deaerated environment specified by ASTM G61. An Ag/AgCl (1 mol/L KCl) reference electrode (RE) was used in place of the saturated calomel electrode due to its compatibility with the MAP-E’s automated handling system, lower maintenance requirements, and reduced toxicity, while still providing stable performance in Cl^−^-containing electrolytes. Additionally, the specimen holder was implemented using the MAP-E’s custom flat-cell design, and tests were performed at room temperature, as opposed to the 25 ° C specified in the standard. While these modifications reflect practical aspects of operating an autonomous, multi-cell platform, the essential elements of the ASTM G61 methodology; electrolyte composition, electrochemical parameters, and interpretation of *E*_pit_, are preserved, enabling a meaningful assessment of the MAP-E’s electrochemical capability.

**Fig. 1:**
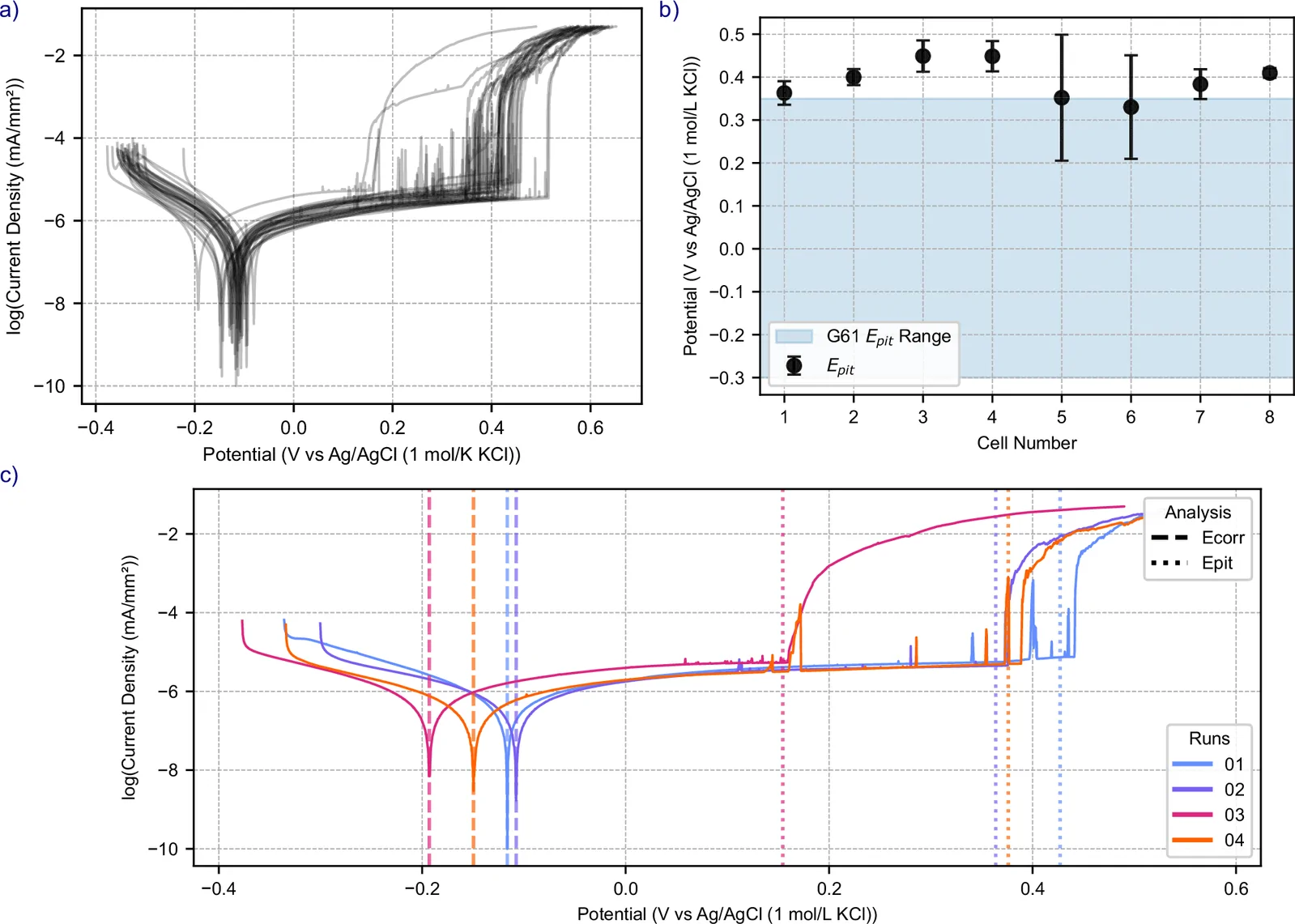
Results from the ASTM G61 replication experiments with the MAP-E. **a** Potentiodynamic polarization curves for the all 304 stainless steel samples (32) in aerated aqueous NaCl solution with mass fraction of 3.56 wt% at room temperature, measured across the eight cells of the MAP-E. **b** Distribution of *E*_pit_ extracted from the polarization curves, showing cell-to-cell variability and overall reproducibility. Error bars indicate standard deviation of measurements within each cell. **c** Polarization curves from just one cell (Cell 6) to illustrate run-to-run stochasticity within a single cell.

All eight REs are calibrated against a common standard before use, and any drift from the standard is corrected for in the data analysis to ensure the results reflect electrochemical differences rather than RE inconsistencies. Figure 1a shows the results from the ASTM G61-analog validation experiments. After a 1 hour rest, PP was performed in aerated 3.56 wt. % NaCl solution at room temperature, at a scan rate of 0.167 mV s^−1^, from − 250 mV vs. *E*_*corr*_ until 5 mA cm^−2^ was reached. Thirty-two 304 stainless steel samples were prepared according to the ASTM procedure and tested across four full the MAP-E runs (8 unique samples per run), rinsing the cell twice with type 1 water between runs to rinse cells and the REs. E_pit_ was extracted using the automated data analysis pipeline described in the adaptive experimentation framework section.

Across all measurements (PP curves available in supplementary information section S1) the mean corrosion potential (*E*_corr_ )was -119 mV ± 21 mV and the mean *E*_pit_ was 392 mV ± 75 mV. The standard deviation of *E*_pit_ across the MAP-E measurements is approximately four times smaller than the inter-laboratory standard deviation implied by ASTM G61 ( ≈ 300 mV, based on a reported 2*σ* spread of ≈ 600 mV across multiple laboratories). At the cell level, variability was even tighter, with standard deviations in most cells < 40 mV, shown in Figure 1b, demonstrating consistent performance in repeated tests within individual cells. The *E*_pit_ values obtained on the MAP-E were consistently higher than those reported in ASTM G61 datasets, with individual measurements up to ≈ 100 mV higher and a mean positive offset of ≈ 40 mV.

The MAP-E results demonstrate high reproducibility and precision, with inter-cell variability below that which was defined in ASTM G61. The elevated average *E*_pit_ and the measurement noise in the passive region are both attributable to testing in aerated electrolyte. Dissolved oxygen increases cathodic reaction kinetics and promotes passive film stabilization, which shifts the pitting potential positively relative to deaerated ASTM conditions^34^. The presence of dissolved oxygen also introduces metastable pitting events that manifest as fluctuations in the current response near *E*_pit_.^35,36^. This behavior is illustrated in Figure 1c, where early pit initiation is observed during two nominally identical runs (3 and 4): in one case the metastable pit transitioned into stable pit growth, whereas in the other it repassivated at approximately the same potential. Notably, these ‘early-pit’ events occur near the mean *E*_pit_ reported in ASTM G61, further supporting the interpretation that the observed differences arise from environmental aeration.

In addition to producing measurement quality consistent with established corrosion test standards, the MAP-E executed all steps of the experiment automatically once samples and stock solutions are setup. In this validation study, eight cells were operated in parallel, enabling all the potentiodynamic tests to be completed with minimal operator involvement beyond routine monitoring over the course of two days. Although the electrochemical runtime remains comparable to conventional testing, operator time was reduced substantially. This capability positions the MAP-E to not only accelerate data generation, but also to support quantitative, data-drive studies.

### Autonomous Stability Diagram

Having established the MAP-E’s reproducibility and precision, we deployed the platform to autonomously generate empirical corrosion stability diagrams. Traditional stability diagrams, such as Pourbaix diagrams, describe the thermodynamic stability of metals^37^, while experimentally generated diagrams relating applied potential to pitting susceptibility, such as Pedeferri diagrams^38,39^, are typically constructed manually and limited to a small number of discrete conditions. The MAP-E extends these concepts by performing PP experiments across pH-Cl^−^ space and selecting subsequent conditions based on a model’s predictive uncertainty. A Gaussian process (GP) surrogate model predicts the *E*_pit_ throughout the design space, and an uncertainty-driven acquisition function guides the platform toward the most informative regions, reducing redundant experiments, as described in the adaptive experimentation framework section. While demonstrated here for 304 stainless steel, this workflow is generalizable, enabling data-driven, autonomous mapping of corrosion susceptibility and understanding of the service envelope of materials across complex environmental conditions.

The exploration space for autonomous corrosion mapping was defined in terms of pH and Cl^−^, two critical variables controlling corrosion behavior for stainless steels and following the concept of Pedeferri diagrams^38,39^. The MAP-E investigated environments spanning pH 3 to 10.5 (0.5-unit increments) and Cl^−^ from 0 mol/L to 0.1 mol/L (semi-logarithmic increments), encompassing 80 unique conditions. Each environment was formulated using a Britton-Robinson (BR)^40^ buffer as the background electrolyte to maintain pH stability while covering a broad range of pH values from a limited set of stock solutions, with controlled additions of sodium chloride stocks achieving the target Cl^−^. A Python-based framework calculates the precise stock volumes to reach the target pH and Cl^−^, after which the liquid handling system automatically prepares each condition and measures the pH in the mixing tank. We observed that the measured pH differ slightly from the target value due to the pumping accuracy but the pH is always measured in the mixing tank prior to dispensing and the measured value that is used for subsequent modeling. Each condition was then characterized electrochemically using the same procedure as the validation measurements, with the automated data extraction workflow described in the adaptive experimentation framework section, generating the *E*_pit_ for the subsequent modeling.

**Fig. 2:**
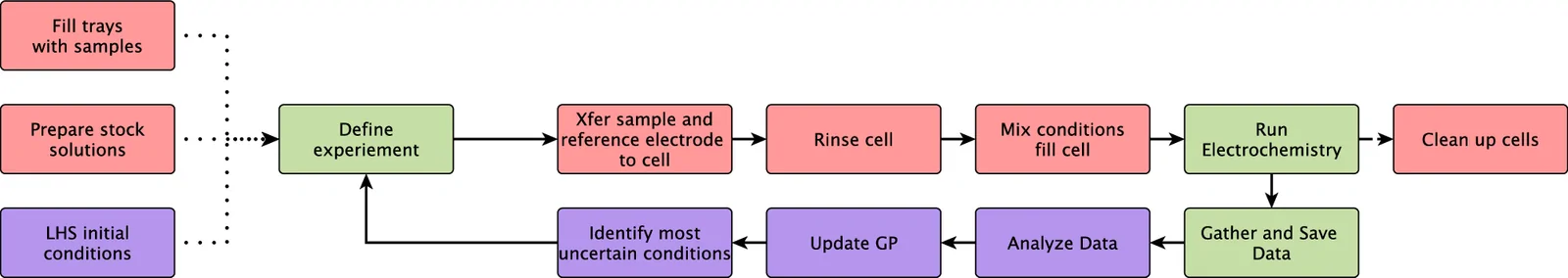
Workflow diagram of the autonomous experimental campaign. High-level workflow diagram of the autonomous experimental workflow for constructing pH-Cl^−^ stability diagrams using the MAP-E platform. The red boxes indicate steps that can primarily be associated with the hardware, the green boxes indicate software-driven steps, and the purple boxes indicate the adaptive modeling components, see methods section for details.

The autonomous experimental workflow is illustrated in Fig. 2. The campaign was initialized with four conditions selected using Latin hypercube sampling (LHS), with duplicate measurements for each condition to capture and model both intrinsic stochasticity in experiments observed in the validation section, and any misidentification of *E*_pit_ from the PP analysis pipeline, discussed in supplementary information section S2. After the initial measurements, the MAP-E proceeded with a fixed budget of 48 additional samples (three trays, 24 conditions in duplicate), autonomously selecting subsequent conditions according to the uncertainty-driven sampling strategy described in the adaptive experimentation framework section. These steps provide a systematic method of exploring environmental space to construct empirical stability diagrams.

**Fig. 3:**
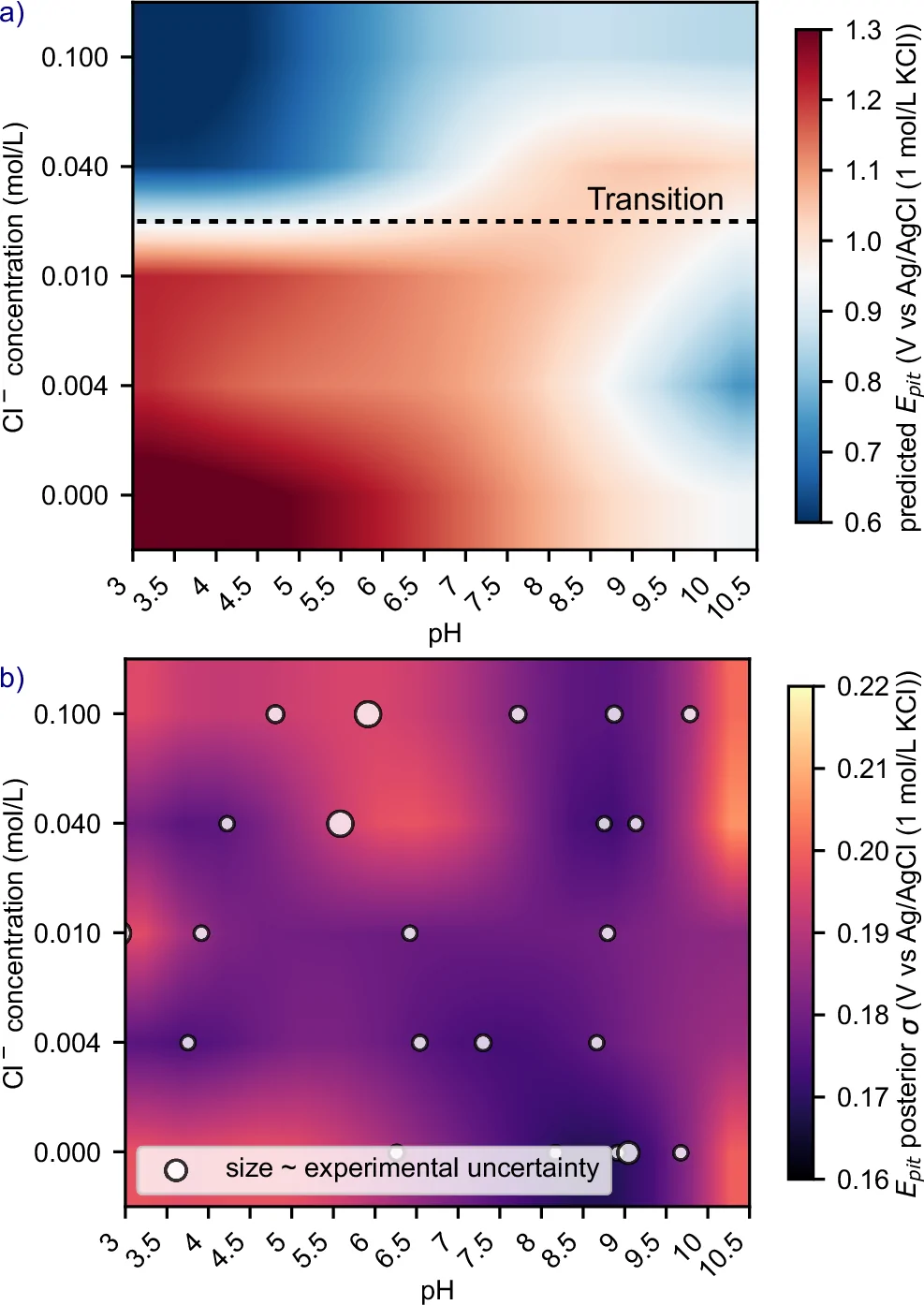
Results from the autonomous stability diagram campaign. **a** Stability diagram showing the predicted *E*_pit_ across the pH-Cl^−^ space for 304 stainless steel, generated autonomously by the MAP-E. **b** Uncertainty map corresponding to the stability diagram, highlighting regions where the model is less certain about *E*_pit_ predictions.

The resulting stability diagram, Fig. 3a, shows that 304 stainless steel is least susceptible to pitting in oxygenated, moderately acidic, low-Cl^−^ environments and most susceptible under high-Cl^−^, acidic conditions. When compared with other conventional mapping studies on 304 stainless steel, the MAP-E-generated stability diagram reproduces the expected decrease in *E*_pit_ with increasing Cl^−^, consistent with increased susceptibility to localized corrosion^41–43^. However, a transition region appears, above which *E*_pit_ decreases with decreasing pH, whereas below this region, the opposite pH dependence is observed. Additionally, all *E*_pit_ values observed in this study are higher ( ≈ 400mV) than those reported in similar mapping studies investigating pitting susceptibility of stainless steel in Cl^−^ environments using conventional solutions^41–45^. The elevated *E*_pit_ values are consistent with the phosphate and borate components of the BR buffer, which have been shown to enhance passive film stability and increase resistance to pitting ^46,47^. These buffering species may also contribute to the observed transition in pH dependence at low Cl^−^ concentrations.

The GP-derived stability diagram resolves this change in behavior across the pH-Cl^−^ space without embedding mechanistic corrosion relationships. At high Cl^−^ concentrations, the observed trends are consistent with established understanding: sufficient Cl^−^ promotes pit initiation, while decreasing pH further destabilizes the passive film and lowers the potential required for pit propagation^48,49^. In these conditions, aggressive Cl^−^ adsorption and reduced oxide stability dominate the pitting response. Below the transition region, Cl^−^ concentrations appear insufficient for Cl^−^-driven pitting processes to dominate the electrochemical response. In this area, the observed variation in *E*_pit_ is likely influenced by the phosphate- and borate-containing buffer chemistry, which promotes passive film stability, and is consistent with published measurements of stainless steel pitting behavior in phosphate- and borate-containing Cl^−^ electrolytes^46,47,50,51^.

The posterior predictive uncertainty map, Figure 3b, highlights regions where additional data would most improve model confidence, with higher uncertainty near the boundaries of the design space where experimental coverage is sparse and where measurements show more variability. Despite these localized regions, the uncertainty surface remains relatively uniform, with an average predictive uncertainty of ≈ 175 mV. Together, these results define the service envelope of 304 stainless steel across the investigated pH-Cl^−^ space and demonstrate that the MAP-E platform can be used in autonomous experimental campaigns.

In this work we present the MAP-E, a modular, HT system capable of autonomously performing parallel electrochemical experiments with individually addressable cells. Validation against a ASTM G61-analog benchmark confirms reproducibility and quantitative precision, with standard deviations in *E*_pit_ approximately one quarter of the inter-laboratory variability reported in the standard, producing data suitable for autonomous studies. Using a posterior-uncertainty-driven experimental framework, the MAP-E autonomously generated empirical pH-Cl^−^ stability diagrams for 304 stainless steel, capturing trends in pitting susceptibility and associated uncertainties. The platform’s architecture enables closed-loop experimentation: selecting, executing, and analyzing experiments without human intervention, while supporting flexible definition of electrochemical workflows beyond corrosion testing. Collectively, these results establish the MAP-E as a generalizable and data-driven tool for mapping corrosion service envelopes, and demonstrate its potential to accelerate both corrosion research and broader electrochemical discovery efforts.

## Methods

The design philosophy of the MAP-E centers on maximizing experimental throughput while minimizing the trade-offs in versatility and flexibility that commonly constrain automated electrochemical platforms. Prior to operation, stock solutions are prepared to span the target environmental range, and polished samples are loaded into the dedicated sample racks. A high-level software interface accepts experiment requests which specify the electrochemical methods, sample selection, and environmental conditions. Once initiated, the MAP-E automatically orchestrates the full workflow. The system loads samples into the electrochemical cells, mixes and dispenses the target electrolyte compositions via the liquid-handling system. It executes the prescribed electrochemical measurements, and processes the resulting data to extract the corrosion parameters of interest while the system rinses the cells and prepares for subsequent experiments. The following sections describe the hardware, control software, and modeling framework for the autonomous stability diagram investigation.

### Hardware

The MAP-E hardware is organized into four subsystems that enable automated, HT electrochemical experiments: (1) the electrochemical cell array, (2) gantry system, (3) liquid handling, and (4) the multi-channel potentiostat.

**Fig. 4:**
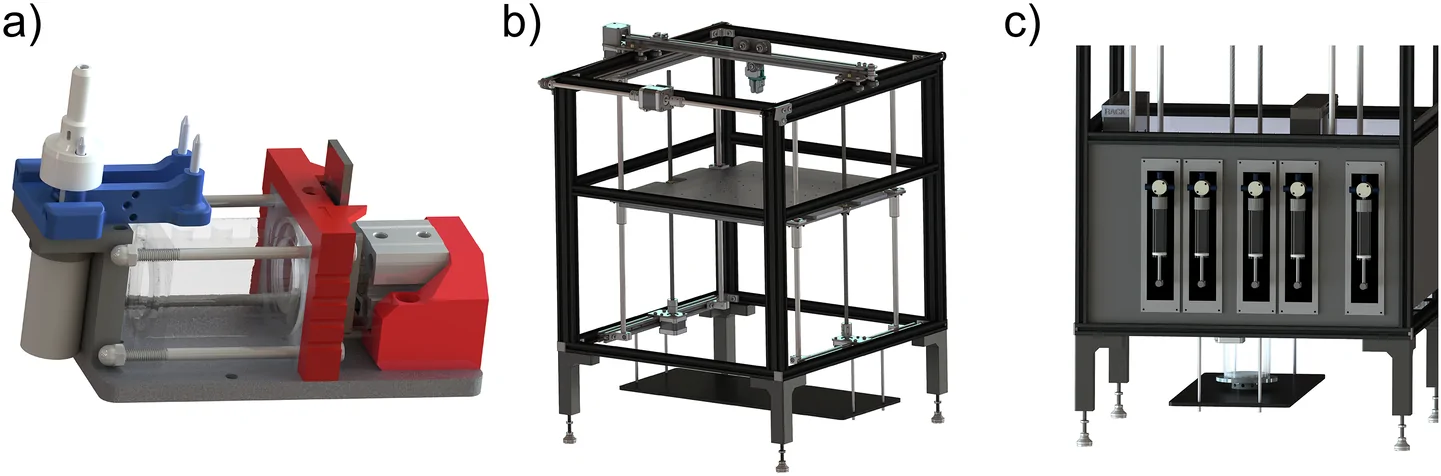
Rendering of the key hardware subsystems of the MAP-E platform. **a** Custom electrochemical flat cell with pneumatic clamping mechanism and integrated electrodes (reference electrode in storage tube on the left, counter electrode mesh in the rear end cap (not visible), and sample as the working electrode clamped by the pneumatic cylinder on the right). **b** Gantry system adapted from a 3D printer. **c** One side of the liquid handling system, showing 5 of the 10 dosing pumps and the central mixing tank attached to the gantry frame, The remaining pumps are mounted on the opposite side of the gantry and are not visible in this rendering.

The electrochemical array consists of eight independent, custom flat cells, constructed from a cylindrical polycarbonate (PC) body with two machined PC end caps. Each cell contains a platinum mesh counter electrode (CE) in the rear end cap, while the front end cap has a 1 cm^2^ hole sealed with a Viton o-ring. A pneumatic cylinder mounted on the front end cap, when triggered, secures a sample against the o-ring. This maintains a 25 mL electrolyte volume that meets the 0.2 mL/mm^2^ minimum ratio recommended by ASTM G31^52^. When not in use, the CH Instruments Ag/AgCl (1 mol/L KCl) REs are stored in a tube mounted outside the cell, shown in Fig. 4a.

A retrofitted 3D printer serves as the Cartesian gantry system and structural backbone for the platform, shown in Figure 4b. The gantry’s platform supports the two sample racks, each accommodating 16 samples (60 mm × 20 mm × 3 mm), and the electrochemical cell array. The gantry is responsible for transferring samples from the racks, as well as moving the REs from their storage tubes into the cells.

Liquid handling is performed by a network of ten 25 mL Tricontinent CX6000 dosing pumps with multi-port valves mounted on the gantry frame, shown in Figure 4c, all connected to a central mixing tank under the gantry equipped with a pH meter and magnetic stir bar. Eight of these pumps are dedicated to delivering electrolyte from the mixing tank to the cells and removing used solution to waste, while the remaining two are used to fill the mixing tank with stock and cleaning solutions, the latter of which can be used to flush cells between experiments.

A Bio-Logic VMP-3 multi-channel potentiostat provides independent electrical control of the eight cells. Connections from the potentiostat are routed through a custom breakout box with gold-plated connectors, ensuring reliable contact. For each cell the electrical connections are made from the breakout box to: the RE through a 2mm banana connector in a 3D-printed holder that enables the gantry to move the RE into position, and easy maintenance; the CE via a fixed ring terminal connecting to a wire attached to the platinum mesh; and the working electrode through a conductive plate at the end of the pneumatic cylinder, which engages the sample upon clamping.

### Control Software

**Fig. 5:**
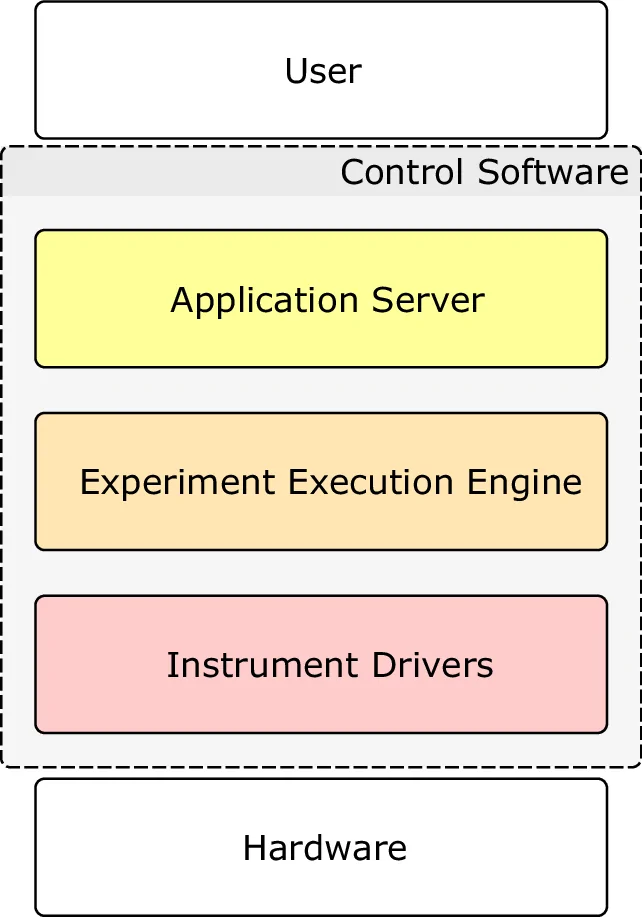
Schematic of the MAP-E software architecture, illustrating the three primary layers: application server, experiment execution engine, and instrument driver libraries.

The MAP-E control software was developed as a modular framework for automated, concurrent experimentation. The architecture comprises three layers: (1) the application server, (2) the experiment execution engine, and (3) the instrument driver libraries, shown in Fig. 5. Together, these layers abstract the underlying hardware complexity and provide a high-level user interface for submitting experiment requests.

**Fig. 6:**
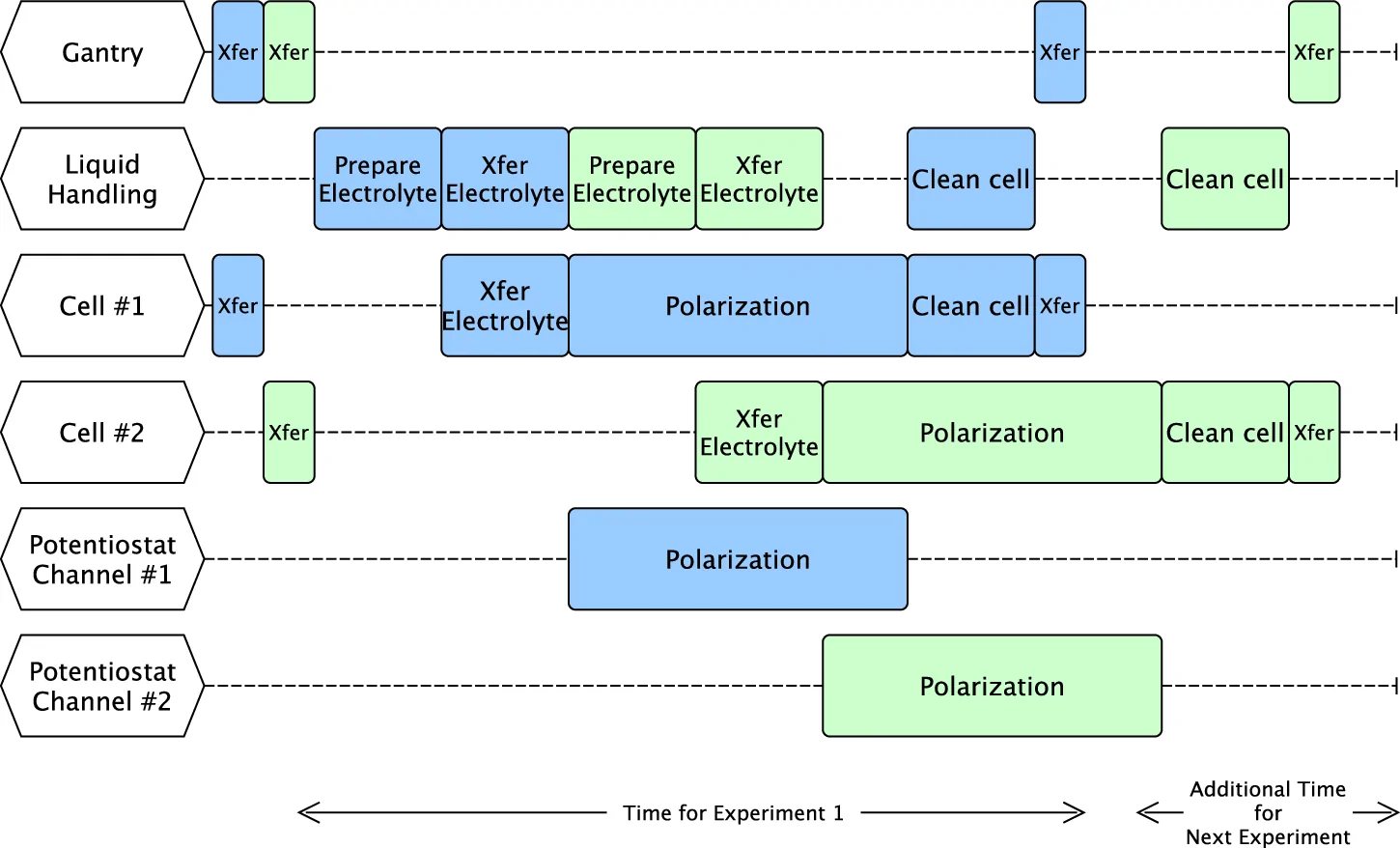
Illustration of the resource allocation and scheduling framework within the experiment execution engine, demonstrating parallel operation of multiple electrochemical cells while managing shared resources like the mixing tank. In this example, separate experiments are denoted by different colors and only two cells are shown for clarity.

The application server provides a network-accessible interface for managing experiment requests and returning the resulting measurement data. The server accepts experiment specifications as structured inputs, handles data collection and storage automatically, as well as maintaining a registry of active and completed jobs to support asynchronous submission, execution, and monitoring. At the core of the control architecture is the experiment execution engine, which orchestrates real-time operation of the hardware subsystems. Experimental procedures are defined as sequences of schedulable tasks (e.g., sample transfer, cell filling, cell cleaning, electrochemical techniques) that are dynamically allocated to available cells or shared resources (e.g., the mixing tank), as illustrated in Fig. 6. This architecture supports fully concurrent operation, allowing multiple experiments to proceed in parallel, while managing shared hardware dependencies transparently. The instrument driver libraries form the interface layer between the execution engine and the physical devices. Each driver exposes the essential functions of its corresponding hardware—such as pumps, actuators, or potentiostat channels—through a consistent software interface, enabling coordination within the broader system. Its modular design allows new instruments or sensors to be incorporated with minimal software modification.

### Adaptive Experimentation Framework

In this work, we perform PP measurements and the resulting data is retrieved from the control software to a user-specified directory. Data is processed automatically, and new experiment requests can then be generated and submitted back to the control software. The raw PP data is analyzed using a Python-based pipeline that identifies the *E*_corr_ and *E*_pit_. To mitigate experimental noise, the logarithm of the current density is smoothed using a Savitzky-Golay filter (second-order polynomial) with respect to potential. To further stabilize the second derivative signal, a rolling mean is applied to the resulting derivative. The *E*_corr_ is identified as the potential corresponding to the minimum absolute current. The *E*_pit_ is determined by locating the maximum of the second derivative ($\frac{{d}^{2}\log (i)}{d{E}^{2}}$) outside of the *E*_corr_ area, corresponding to the onset of stable pitting. These parameters are stored and made available for subsequent modeling.

A GP regression model serves as the surrogate model for predicting *E*_pit_ across the design space. The GP employs a scaled Matérn covariance kernel (*ν* = 2.5) with automatic relevance determination to capture varying correlation length scales in each input dimension. A heteroscedastic likelihood is used to represent measurement-dependent noise, accounting for variability between duplicate experiments. The model is initialized with four LHS conditions and iteratively updated as new data are collected. Experimental selection is guided by an uncertainty-driven acquisition function that prioritizes conditions with the highest posterior predictive variance in *E*_pit_. This approach directs the MAP-E toward regions of high model uncertainty or experimental disagreement, balancing exploration of untested conditions with refinement of noisy regions.

### Materials and Sample Preparation

Standard Type 304 stainless steel (UNS S30403) sheet stock was procured from McMaster-Carr, water-jet cut to size, and polished to 600-grit following ASTM G61. For the validation study, a 3.56 wt. % sodium chloride solution was prepared according to ASTM G61^33^. For the autonomous stability diagram experiments, stock solutions for the BR buffer^40^ were prepared using reagent-grade chemicals at the following concentrations: sodium hydroxide (0.3 mol/L), phosphoric acid (0.2 mol/L), acetic acid (0.2 mol/L), and boric acid (0.2 mol/L). Two sodium chloride stock solutions were also prepared to span the Cl^−^ range: 0.04 mol/L and 0.4 mol/L. All solutions were made with type 1 water (resistivity > 18 mol/L*Ω* ⋅ cm^2^ at 25 °C) and mixed to generate the desired electrolyte compositions for their respective experiments.

## Supplementary information

Supplementary information

## Data Availability

The data generated by the MAP-E during the validation and autonomous stability diagram experiments, including raw electrochemical measurements, processed corrosion parameters, and the resulting stability diagrams, are available at the following GitHub repository.

## Code availability

The code the to plot the data, the GP surrogate modeling, and the adaptive experimental framework is available at the following GitHub repository.
