# Supplementary material for: Materials Acceleration Platform for Electrochemistry: a Platform for Autonomous Electrochemistry
Source: Npj Mater Degrad. 2026 Jun 4;10(1):111. doi: 10.1038/s41529-026-00822-8 (PMC13553302; doi:10.1038/s41529-026-00822-8)
Supplement: Supplementary file 1 [file 41529_2026_822_MOESM1_ESM.pdf]

# **Supplementary information for:**
# Materials Acceleration Platform for Electrochemistry: a Platform for Autonomous Electrochemistry

Daniel Persaud[1,2], Mike Werezak[1], Mark Xu[1], Melyne Zhou[2], Frank Benkel[1], Xin Peng[1], Vahid Attari[1], Brian DeCost[3], Ashley Dale[2], Nicholas Senior[1], Gabriel Birsan[1, *], and Jason Hattrick-Simpers[1, 2, 4, 5, 6, **]

[1]*CanmetMATERIALS, Natural Resources Canada, 183 Longwood Road South, Hamilton, ON, Canada*
[2]*Department of Materials Science and Engineering, University of Toronto, 27 King's College Cir, Toronto, ON, Canada*
[3]*Material Measurement Laboratory, National Institute of Standards and Technology, 100 Bureau Dr, Gaithersburg, MD, USA*
[4]*Acceleration Consortium, University of Toronto, 27 King's College Cir, Toronto, ON, Canada.*
[5]*Vector Institute for Artificial Intelligence, 661 University Ave, Toronto, ON, Canada.*
[6]*Schwartz Reisman Institute for Technology and Society, 101 College St, Toronto, ON, Canada.*
[*]*Correspondence: gabriel.birsan@NRCan-RNCan.gc.ca*
[**]*Correspondence: jason.hattrick.simpers@utoronto.ca*

## S1 Corrosion Validation Potentiodynamic Polarization

Below are the 32 potentiodynamic polarization (PP) scans and automated analysis to extract the corrosion potential ($E_{\mathrm{corr}}$) and pitting potential ($E_{\mathrm{pit}}$), for each of the ASTM G61-analog experiments performed to validate the MAP-E. The $E_{\mathrm{pit}}$ values extracted from the automated analysis closely match the onset of stable pitting observed in the raw PP scans, all within $\approx 50mV$.

**Potentodynamic Polarization scans for batch 1 of replication experiments with the MAP-E.**

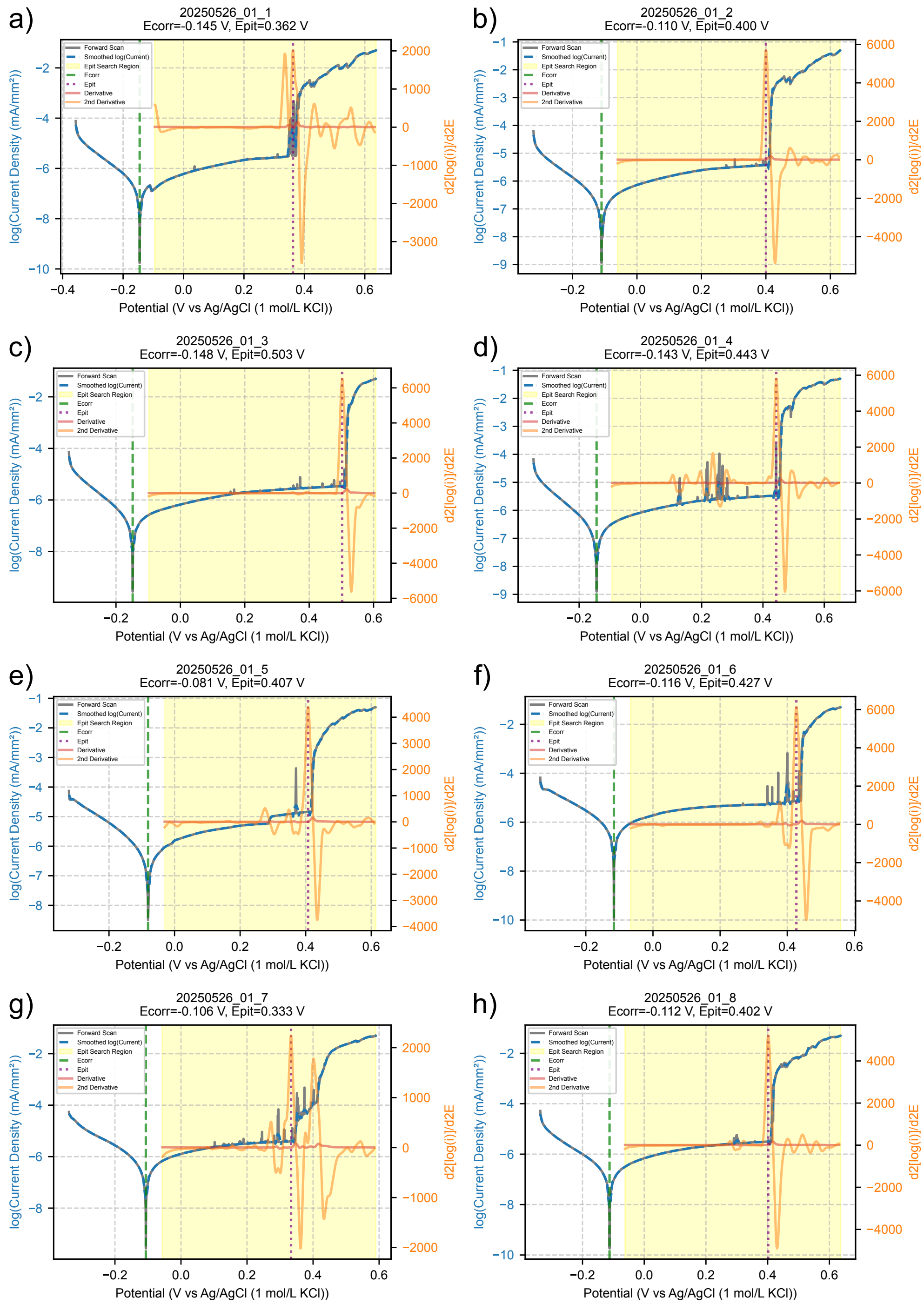


Figure S1: a) cell 1, b) cell 2, c) cell 3, d) cell 4, e) cell 5, f) cell 6, g) cell 7, h) cell 8. Each plot shows the logarithm of the current density vs. potential. The solid grey line is the raw data, blue dashed line is the smoothed data, solid red line is the smoothed data derivative, solid orange line is the smoothed data second derivative, dashed green line is the extracted $E_{\mathrm{corr}}$, and dotted purple line is the extracted $E_{\mathrm{pit}}$.

**Potentodynamic Polarization scans for batch 2 of replication experiments with the MAP-E.**

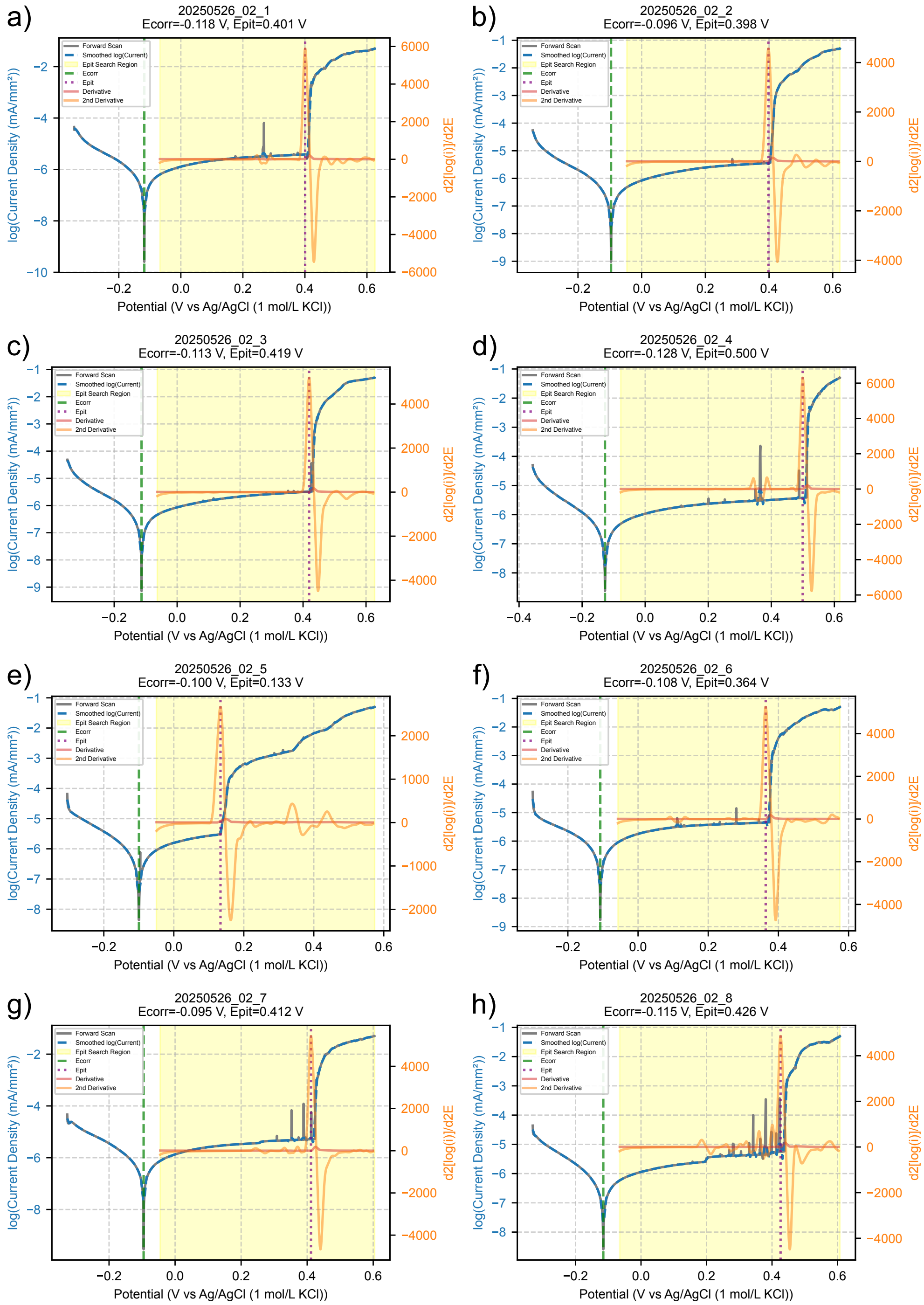


Figure S2: a) cell 1, b) cell 2, c) cell 3, d) cell 4, e) cell 5, f) cell 6, g) cell 7, h) cell 8. Each plot shows the logarithm of the current density vs. potential. The solid grey line is the raw data, blue dashed line is the smoothed data, solid red line is the smoothed data derivative, solid orange line is the smoothed data second derivative, dashed green line is the extracted $E_{\mathrm{corr}}$, and dotted purple line is the extracted $E_{\mathrm{pit}}$.

**Potentodynamic Polarization scans for batch 3 of replication experiments with the MAP-E.**

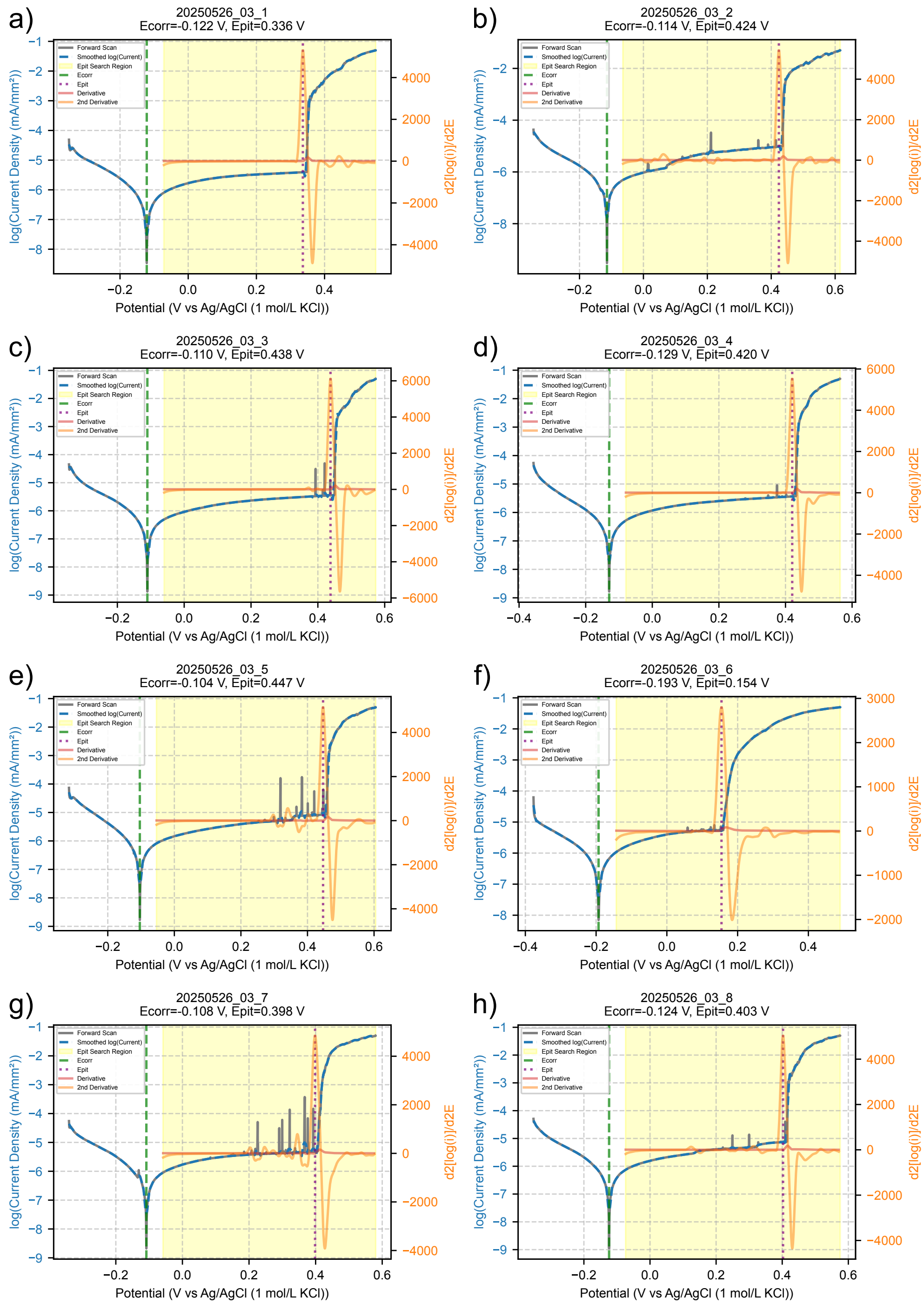


Figure S3: a) cell 1, b) cell 2, c) cell 3, d) cell 4, e) cell 5, f) cell 6, g) cell 7, h) cell 8. Each plot shows the logarithm of the current density vs. potential. The solid grey line is the raw data, blue dashed line is the smoothed data, solid red line is the smoothed data derivative, solid orange line is the smoothed data second derivative, dashed green line is the extracted $E_{\mathrm{corr}}$, and dotted purple line is the extracted $E_{\mathrm{pit}}$.

**Potentodynamic Polarization scans for batch 4 of replication experiments with the MAP-E.**

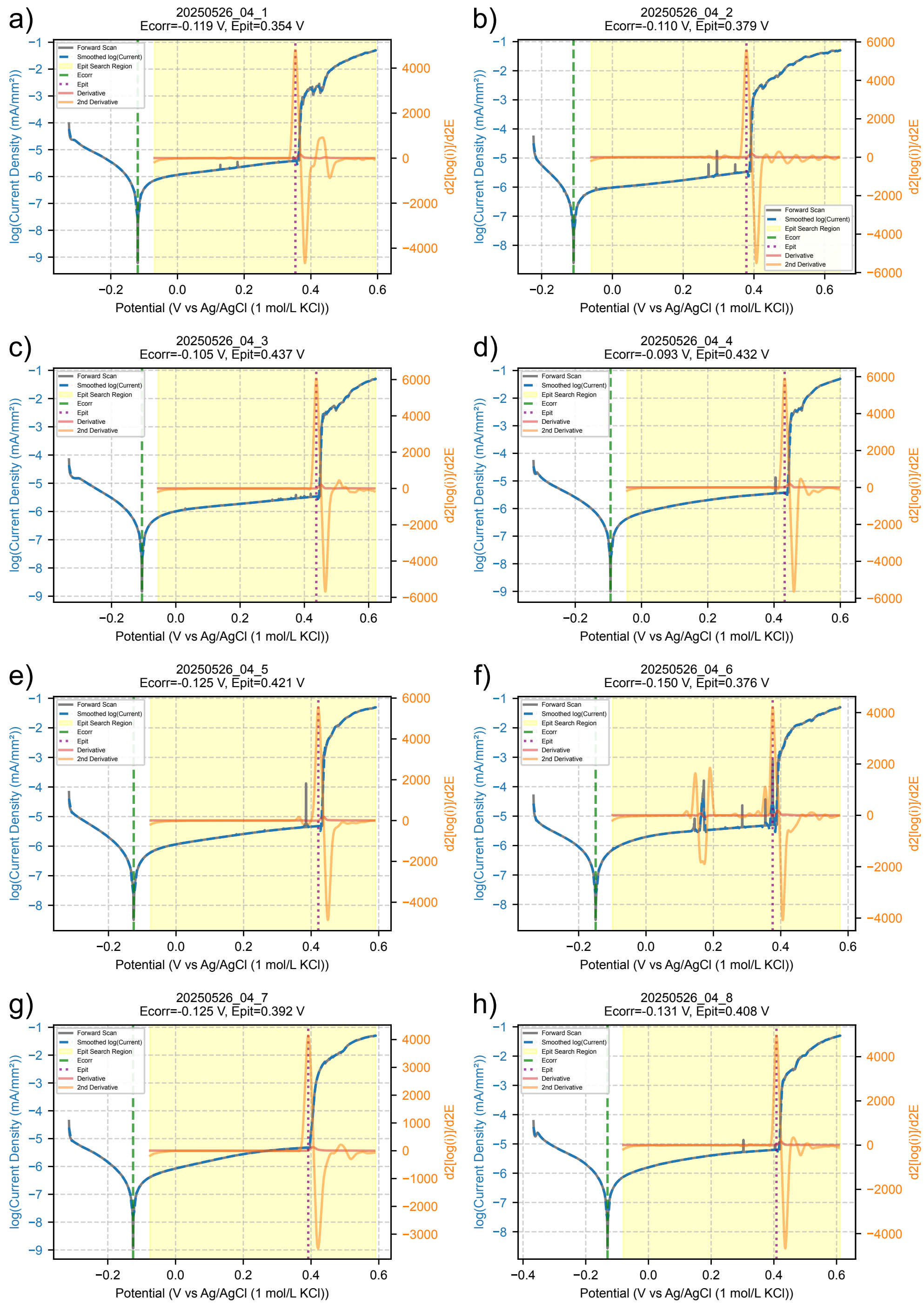


Figure S4: a) cell 1, b) cell 2, c) cell 3, d) cell 4, e) cell 5, f) cell 6, g) cell 7, h) cell 8. Each plot shows the logarithm of the current density vs. potential. The solid grey line is the raw data, blue dashed line is the smoothed data, solid red line is the smoothed data derivative, solid orange line is the smoothed data second derivative, dashed green line is the extracted $E_{\mathrm{corr}}$, and dotted purple line is the extracted $E_{\mathrm{pit}}$.

## Potentodynamic Polarization scans for replication experiments with the MAP-E grouped by cell.

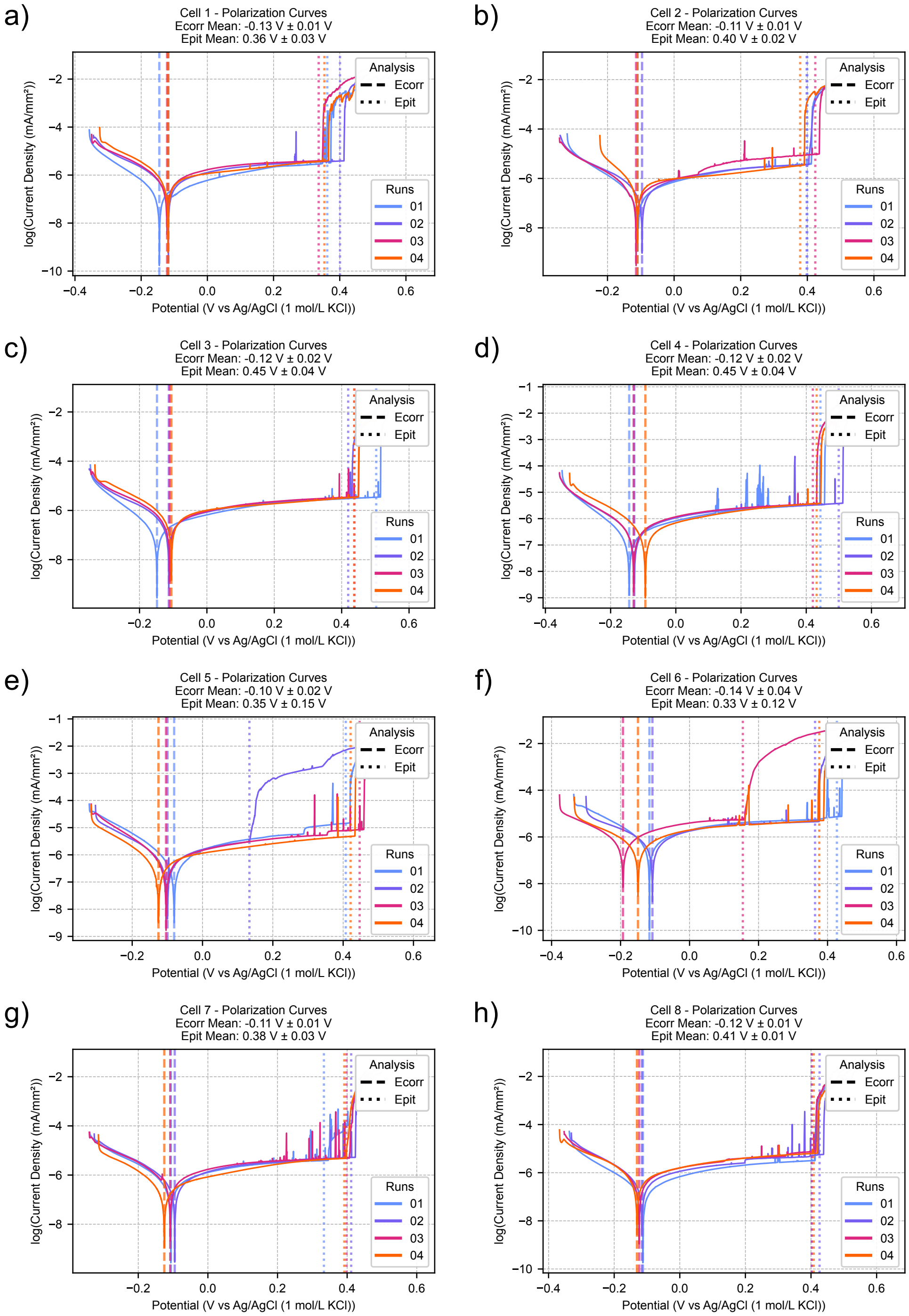


Figure S5: a) cell 1, b) cell 2, c) cell 3, d) cell 4, e) cell 5, f) cell 6, g) cell 7, h) cell 8. Each plot shows the logarithm of the current density vs. potential. The blue solid line is scan 1, purple solid line is scan 2, pink solid line is scan 3, and orange solid line is scan 4. The dashed lines are the $E_{corr}$ and dotted lines are the $E_{pit}$ for each scan, with the same color coding.

## S2 Autonomous Stability Diagram Potentodynamic Polarization

Below are all potentodynamic polarization (PP) scans and automated analyses used to construct the autonomous stability diagram shown in Figure 3 of the main text, including the 4 initial conditions (8 scans total) and 6 iterations of adaptive experimentation (4 conditions per round, 2 scans per condition), for a total of 56 PP scans.

Similar to the validation experiments in section S1, the automated $E_{\mathrm{pit}}$ extraction closely matched the visually identifiable onset of stable pitting in the majority of cases, with 85% of extracted values falling within approximately 50 mV of manual interpretation.

In a small number of cases, the automated analysis identified noise fluctuations or secondary current increases instead of the primary pitting transition. Specifically, two scans:

- 20250922_004_6
- 20250922_004_7

exhibited noise-related misidentification, while six additional scans:

- 20250812_004
- 20250903_002
- 20250916_004

corresponded to either secondary current rises or scans where stable pitting was not clearly observed.

These behaviors represent known limitations of the current automated extraction algorithm. However, they did not significantly affect the adaptive experimentation workflow because duplicate measurements were performed at each condition and disagreement between replicates was incorporated into the uncertainty estimation used by the GP model. Overall, the extracted values remained sufficient to capture the dominant trends in the stability diagram.

## Potentodynamic Polarization scans for iteration 0 of stability diagram building

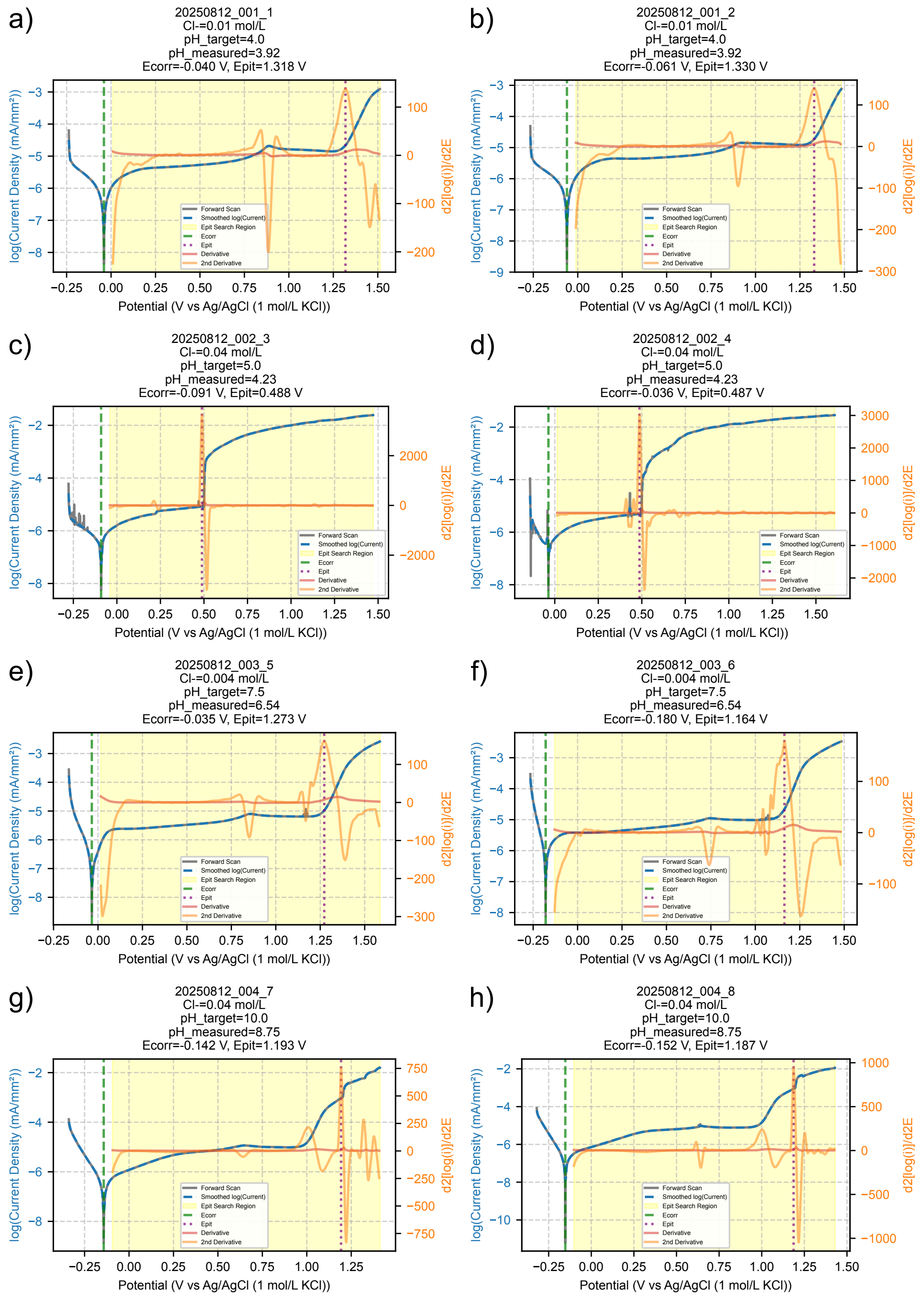


Figure S6: a) cell 1, b) cell 2, c) cell 3, d) cell 4, e) cell 5, f) cell 6, g) cell 7, h) cell 8. Each plot shows the logarithm of the current density vs. potential. The solid grey line is the raw data, blue dashed line is the smoothed data, solid red line is the smoothed data derivative, solid orange line is the smoothed data second derivative, dashed green line is the extracted $E_{\mathrm{corr}}$, and dotted purple line is the extracted $E_{\mathrm{pit}}$.

## Potentodynamic Polarization scans for iteration 1 of stability diagram building

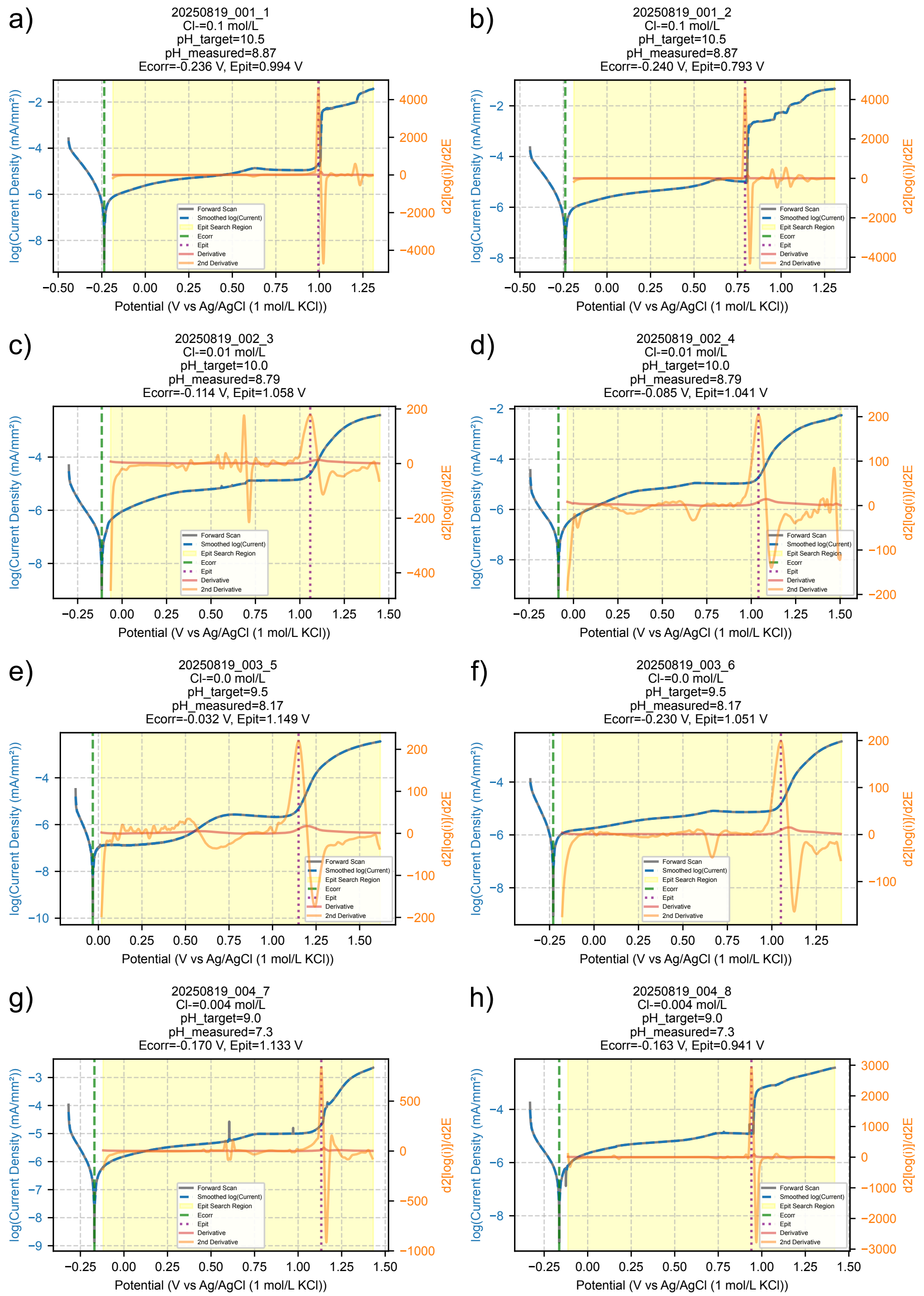


Figure S7: a) cell 1, b) cell 2, c) cell 3, d) cell 4, e) cell 5, f) cell 6, g) cell 7, h) cell 8. Each plot shows the logarithm of the current density vs. potential. The solid grey line is the raw data, blue dashed line is the smoothed data, solid red line is the smoothed data derivative, solid orange line is the smoothed data second derivative, dashed green line is the extracted $E_{\mathrm{corr}}$, and dotted purple line is the extracted $E_{\mathrm{pit}}$.

## Potentodynamic Polarization scans for iteration 2 of stability diagram building

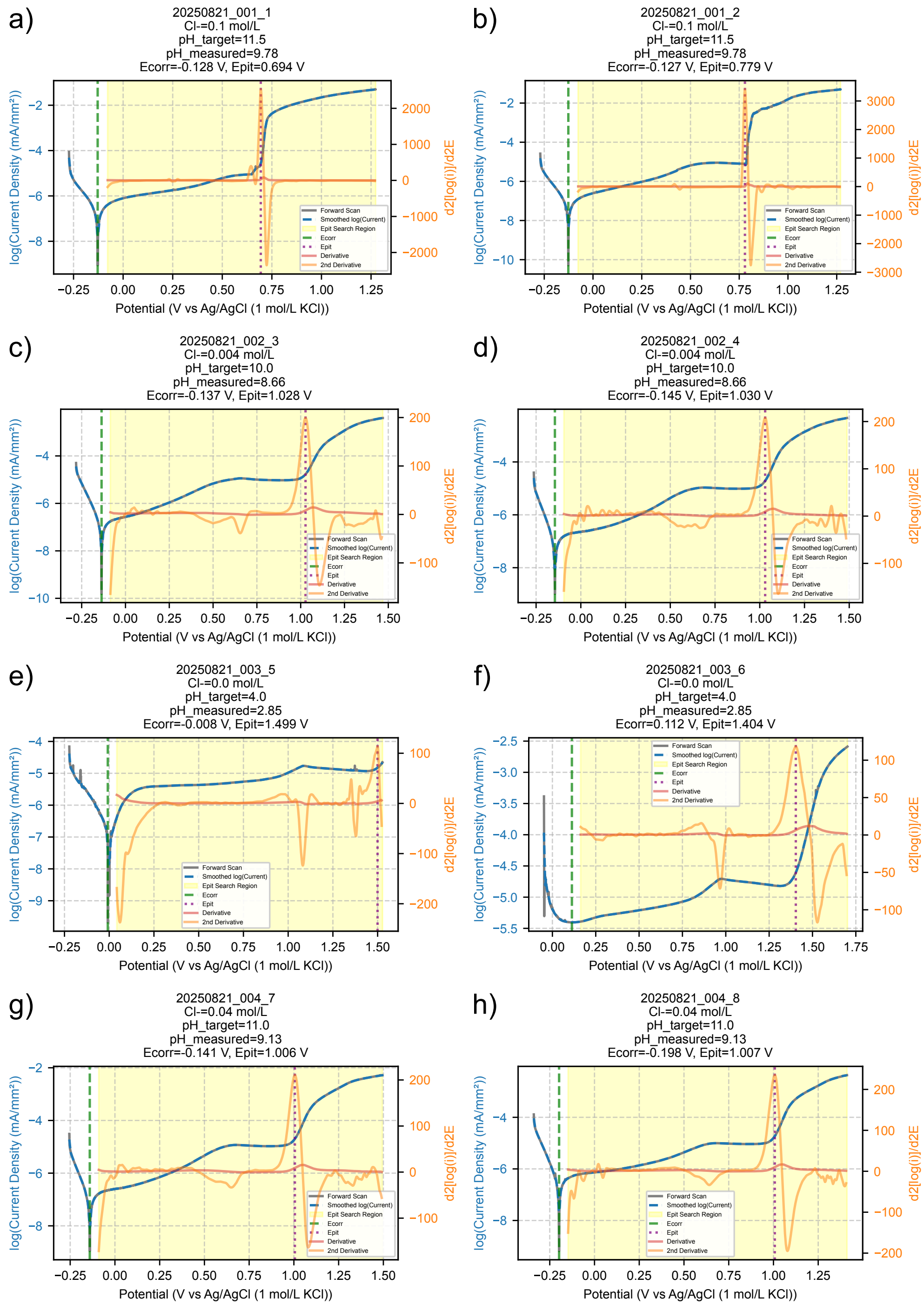


Figure S8: a) cell 1, b) cell 2, c) cell 3, d) cell 4, e) cell 5, f) cell 6, g) cell 7, h) cell 8. Each plot shows the logarithm of the current density vs. potential. The solid grey line is the raw data, blue dashed line is the smoothed data, solid red line is the smoothed data derivative, solid orange line is the smoothed data second derivative, dashed green line is the extracted $E_{\mathrm{corr}}$, and dotted purple line is the extracted $E_{\mathrm{pit}}$.

## Potentodynamic Polarization scans for iteration 3 of stability diagram building

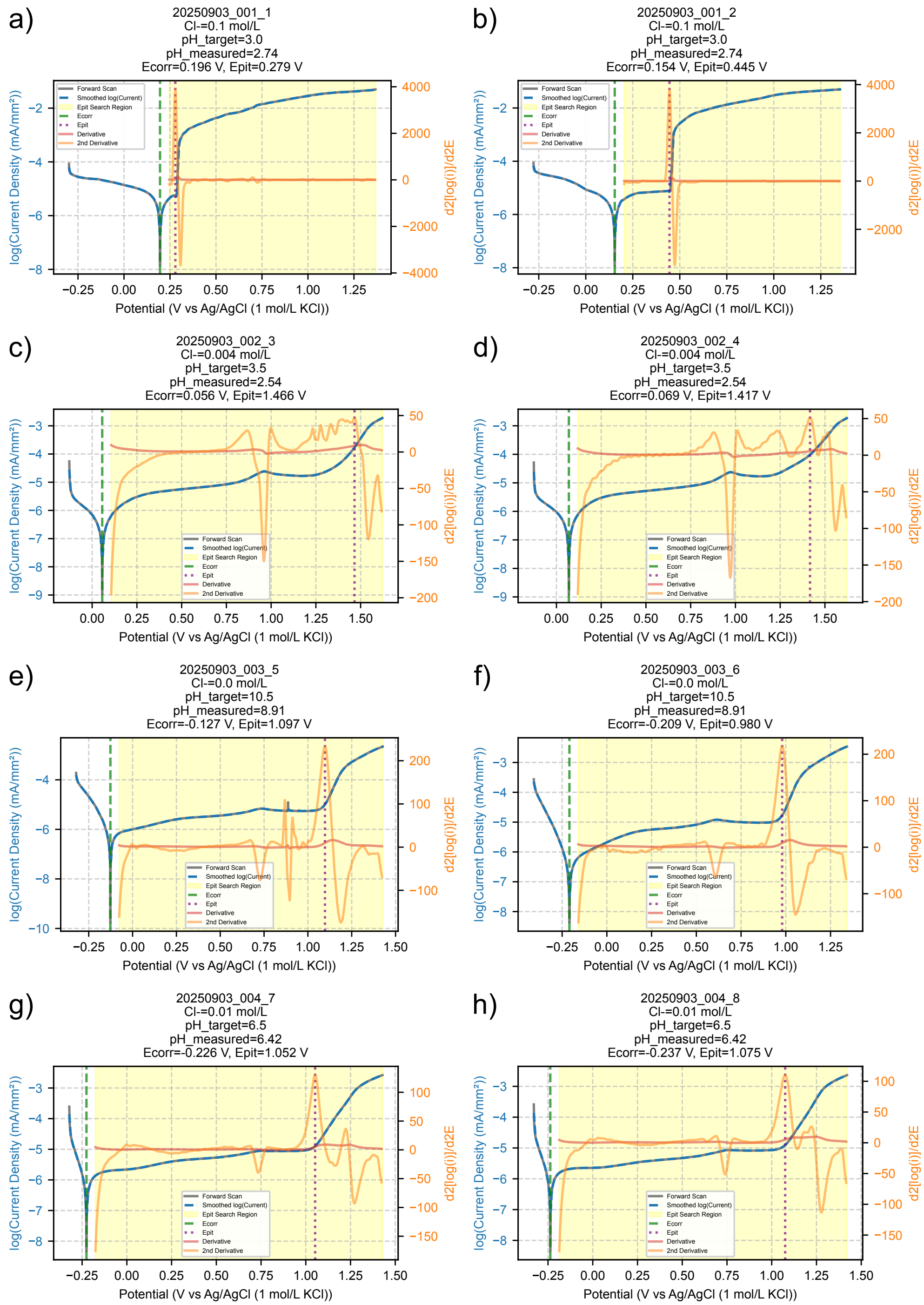


Figure S9: a) cell 1, b) cell 2, c) cell 3, d) cell 4, e) cell 5, f) cell 6, g) cell 7, h) cell 8. Each plot shows the logarithm of the current density vs. potential. The solid grey line is the raw data, blue dashed line is the smoothed data, solid red line is the smoothed data derivative, solid orange line is the smoothed data second derivative, dashed green line is the extracted $E_{\mathrm{corr}}$, and dotted purple line is the extracted $E_{\mathrm{pit}}$.

## Potentodynamic Polarization scans for iteration 4 of stability diagram building

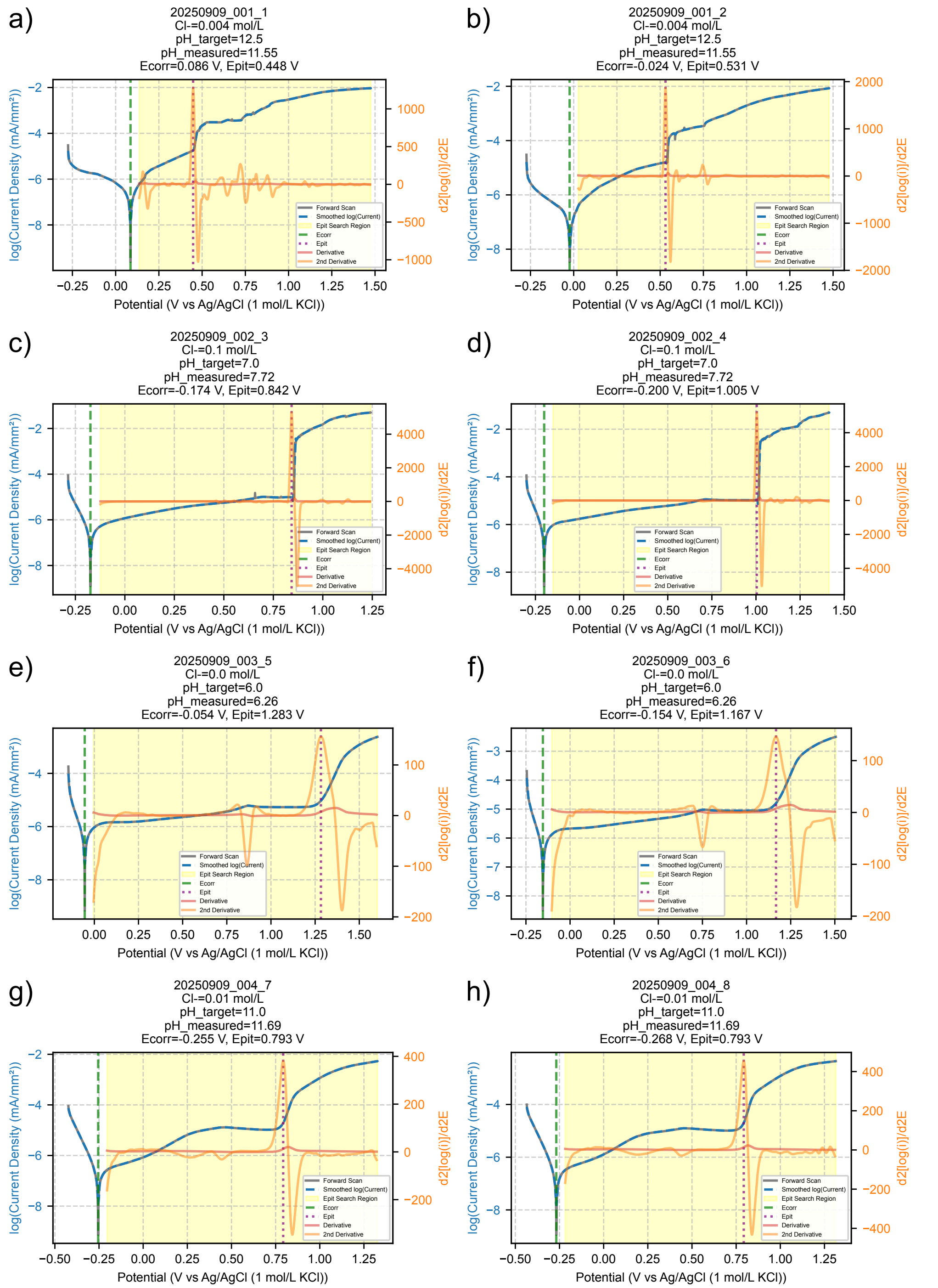


Figure S10: a) cell 1, b) cell 2, c) cell 3, d) cell 4, e) cell 5, f) cell 6, g) cell 7, h) cell 8. Each plot shows the logarithm of the current density vs. potential. The solid grey line is the raw data, blue dashed line is the smoothed data, solid red line is the smoothed data derivative, solid orange line is the smoothed data second derivative, dashed green line is the extracted $E_{\mathrm{corr}}$, and dotted purple line is the extracted $E_{\mathrm{pit}}$.

## Potentodynamic Polarization scans for iteration 5 of stability diagram building

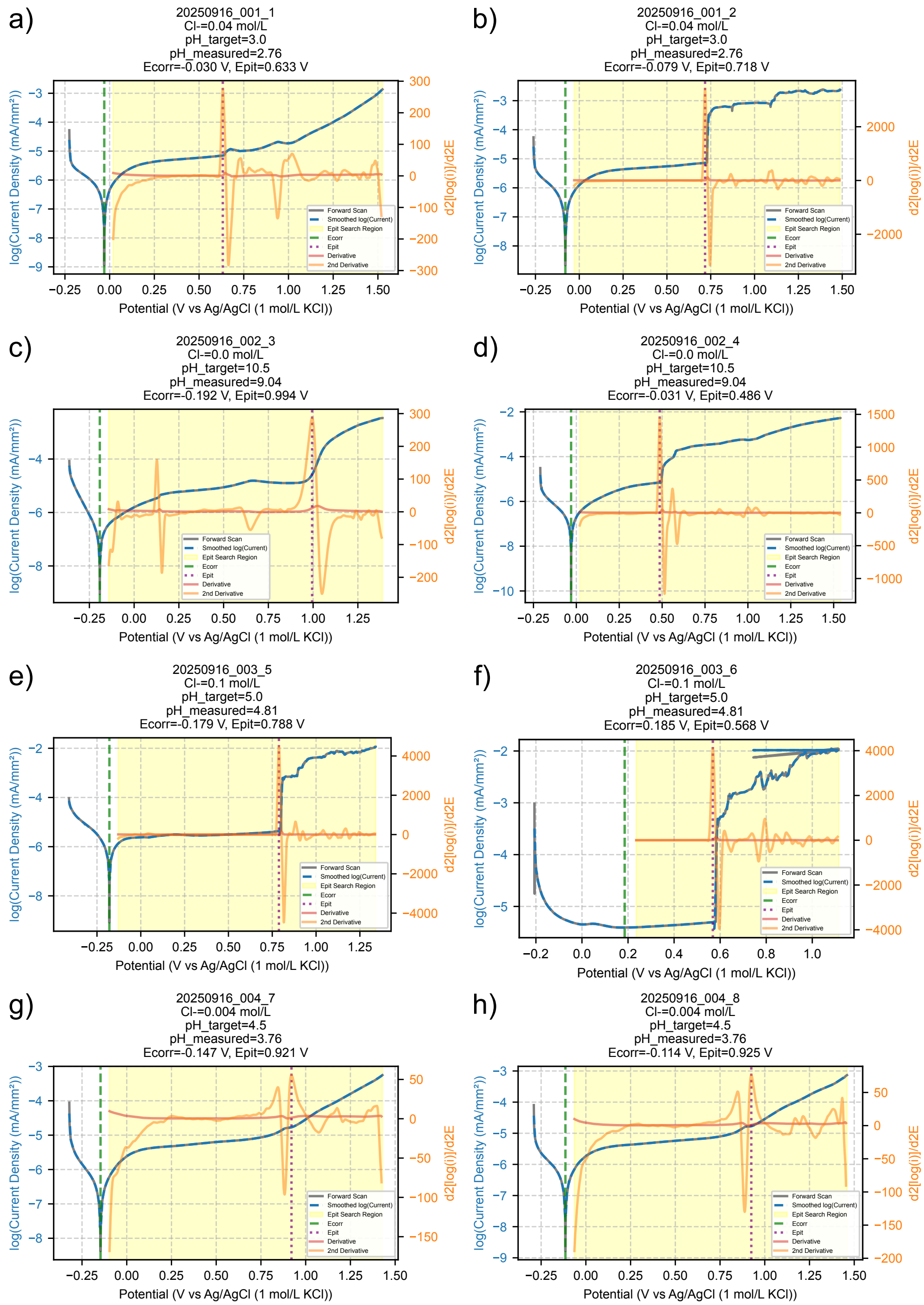


Figure S11: a) cell 1, b) cell 2, c) cell 3, d) cell 4, e) cell 5, f) cell 6, g) cell 7, h) cell 8. Each plot shows the logarithm of the current density vs. potential. The solid grey line is the raw data, blue dashed line is the smoothed data, solid red line is the smoothed data derivative, solid orange line is the smoothed data second derivative, dashed green line is the extracted $E_{\mathrm{corr}}$, and dotted purple line is the extracted $E_{\mathrm{pit}}$.

## Potentodynamic Polarization scans for iteration 6 of stability diagram building

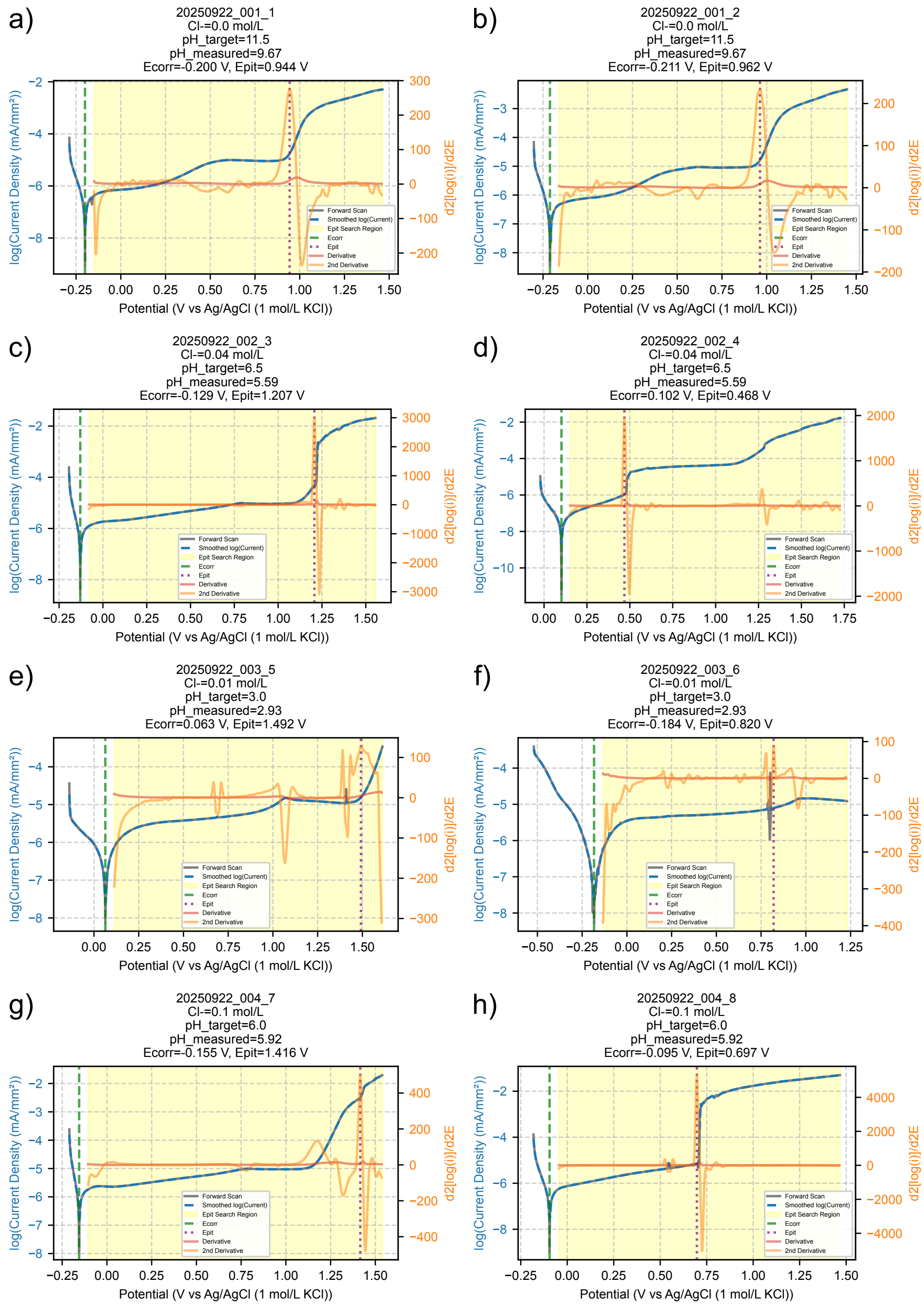


Figure S12: a) cell 1, b) cell 2, c) cell 3, d) cell 4, e) cell 5, f) cell 6, g) cell 7, h) cell 8. Each plot shows the logarithm of the current density vs. potential. The solid grey line is the raw data, blue dashed line is the smoothed data, solid red line is the smoothed data derivative, solid orange line is the smoothed data second derivative, dashed green line is the extracted $E_{\mathrm{corr}}$, and dotted purple line is the extracted $E_{\mathrm{pit}}$.

## S3 Autonomous Stability Diagram Chloride dependence

Below is all the $E_{\mathrm{pit}}$ values plotted against pH for the 56 PP scans performed as part of the autonomous stability diagram experiment, grouped by $Cl^-$. The traces corresponding to 0.04 mol/L and 0.1 mol/L $Cl^-$ show a positive correlation between pH and $E_{\mathrm{pit}}$, while the traces corresponding to 0 mol/L, 0.004 mol/L, and 0.01 mol/L $Cl^-$ show a negative correlation between pH and $E_{\mathrm{pit}}$.

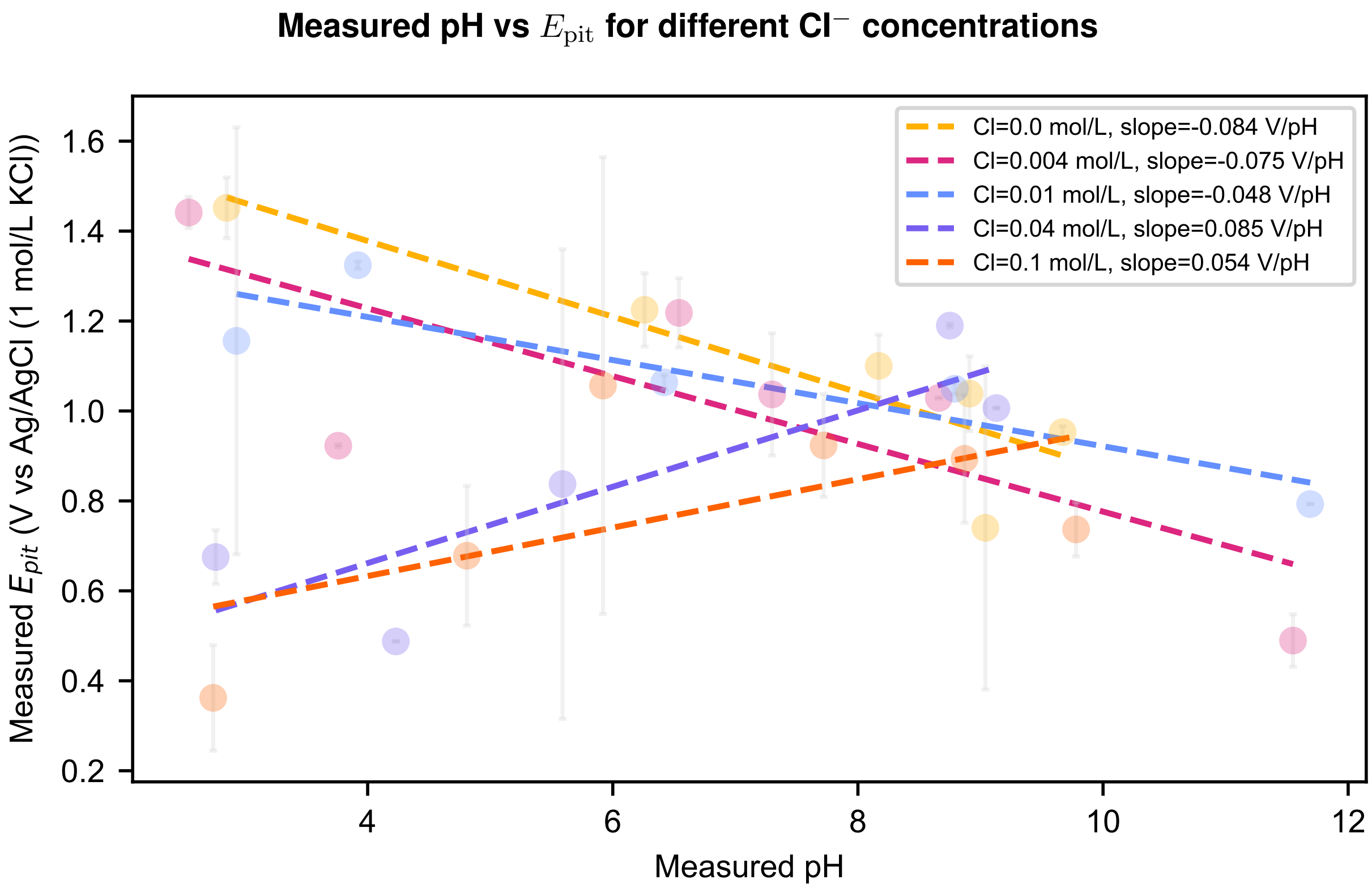


Figure S13: Scatter plot of measured pH vs. $E_{\mathrm{pit}}$ for the 56 PP scans performed as part of the autonomous stability diagram experiment, grouped by $Cl^-$ concentration, with 0 mol/L in yellow, 0.004 mol/L in pink, 0.01 mol/L in blue, 0.04 mol/L in purple, and 0.1 mol/L in orange.
